# The Production and Characterization of an Aminolyzed Polyhydroxyalkanoate Membrane and Its Cytocompatibility with Osteoblasts

**DOI:** 10.3390/molecules30040950

**Published:** 2025-02-18

**Authors:** Qiulan Luo, Fuming Zou, Dongjuan Yang, Yongping Huang, Dajie Xian, Ying Nie, Zhenxia Zhang, Yuzhong Zheng, Yaqun Liu, Fei Zhou, Peikui Yang, Yuting Jiang, Xianjing Huang, Xianghui Zou

**Affiliations:** Guangdong Provincial Key Laboratory of Functional Substances in Medicinal Edible Resources and Healthcare Products, School of Life Sciences and Food Engineering, Hanshan Normal University, Chaozhou 521041, China; 2521@hstc.edu.cn (Q.L.); 14758591618@163.com (F.Z.); dongjy@hstc.edu.cn (D.Y.); 20190001@hstc.edu.cn (Y.H.); 15916449694@163.com (D.X.); 20190030@hstc.edu.cn (Y.N.); zzx8411@hstc.edu.cn (Z.Z.); zhengyuzhong@gmail.com (Y.Z.); 20200048@hstc.edu.cn (Y.L.); zhoufei@hstc.edu.cn (F.Z.); peikyang@hstc.edu.cn (P.Y.); 15876145248@163.com (Y.J.); tina247478462@outlook.com (X.H.)

**Keywords:** biopolyester film, poly-3-hydroxybutyrate-co-3-hydroxyhexanoate, ammonolysis reaction, osteoblast, cytocompatibility

## Abstract

Polyhydroxyalkanoates (PHAs), recognized as a medical biomaterial, have been proven to promote cell proliferation and tissue repair. PHA has a variety of forms: PHB, PHV, PHHx, and PHBHHx, etc. In this study, PHBHHx was selected as the precursor to fabricate biopolyester films. Specifically, a novel type of biopolyester film was generated through an ammonolysis cross-linking reaction in conjunction with polyamidoamine dendrimer G2.0 (PAMAM). The properties of the resultant biopolyester films were comprehensively evaluated, encompassing surface characteristics, amino group content, and water contact angle. The drug-loading properties and compatibility with osteoblasts of the biopolyester films were also determined. The findings revealed that following aminolysis, the biopolyester film surface exhibited enhanced roughness and an enlarged surface area. Moreover, as the aminolysis duration extended, the hydrophilicity and drug-loading efficiency were significantly augmented. Post-aminolysis, the PHBHHx membrane exhibited a more favorable environment for the adhesion and proliferation of osteoblasts. Overall, the biopolyester film developed in this study provides novel insights and materials for tissue engineering, especially bone tissue repair.

## 1. Introduction

Polyhydroxyalkanoates (PHAs), a class of polymeric biopolyester, are synthesized by microorganisms and function as a carbon and energy source in cells [1]. Ever since their first emergence as a sustainable substitute for traditional petrochemical-based polymers [2], PHAs have attracted increasing interest. Based on the number of carbon atoms in the PHA monomer, PHAs can be classified into two categories [3]. One is short-chain PHAs (with 3–5 carbon atoms in the monomer), such as poly-3-hydroxybutyrate (PHB) and poly-3-hydroxyvalerate (PHV). The other is medium- and long-chain PHAs (with six or more carbon atoms in the monomer), such as poly-3-hydroxyhexanoate (PHHx). In recent years, copolymers composed of short-chain and medium- and long-chain monomers have been developed, such as poly-3-hydroxybutyrate-co-3-hydroxyhexanoate (PHBHHx) [4], which exhibits enhanced toughness and elasticity in comparison to traditional PHAs.

Recently, tissue engineering has primarily been used to simulate the in vitro physiological environment. Normal tissue cells cultured in vitro are adhered to biocompatible and biodegradable biological materials and supplied with nutrients to foster cell population expansion [5]. The formation of scaffold complexes, such as fibrous scaffolds, can provide an extracellular matrix (ECM)-like structure and a desired network for cell growth [6]. These complexes can then be implanted into body tissue or the damaged part of an organ, ultimately aiming to repair or improve the function of the damaged tissue or organ. Concurrently, due to their outstanding attributes, including thermoplastic processability, adjustable mechanical properties, and biocompatibility and biodegradability, PHA materials have been harnessed as polymeric substances [7,8], especially as tissue engineering materials. PHA materials have been successfully applied in diverse areas of tissue engineering, such as for an artificial esophagus [9], nerve regeneration conduits [10], artificial cartilage [11], and artificial blood vessels [12]. Their utilization has led to remarkable achievements in spinal cord repair, skin repair, pancreatic cell migration, and cartilage tissue regeneration.

Among various PHA biopolyester films, 3D scaffolds fabricated from materials such as PHB, poly-3-hydroxybutyrate-co-3-hydroxyvalerate (PHBV), poly-(R)-3-hydroxybutyrate-co-4-hydroxybutyrate (P3HB4HB), and PHBHHx have been considered as safe and innocuous [13]. These scaffolds possess a strong affinity for osteoblasts and are non-carcinogenic. Electrospun PHBV fibers and films have demonstrated significant potential in facilitating the adhesion of osteoblasts and fibroblasts [14]. Human fetal osteoblasts cultured on diverse nanofibrous scaffolds containing PHBV manifested cell proliferation and alkaline phosphatase activity [15]. The PHA extracted from microbial granules displays excellent tensile strength, thermoplasticity, and elastomeric characteristics, which renders it highly suitable for bone scaffold fabrication. Extensive investigations have been carried out regarding the biocompatibility between poly (3-hydroxybutyrate) (PHB) and osteoblasts. Collectively, PHAs (encompassing PHB and PHBV, among others) hold great promise in the realm of bone tissue engineering applications.

However, prior research efforts have revealed that, despite the fact that PHBHHx boasts favorable film-forming capabilities, its compatibility with osteoblasts remains subpar, thereby constraining its application in bone tissue repair procedures. Current works are primarily focused on modifying it with biomacromolecules. Remarkably, the modified biopolyester film has exhibited a substantial enhancement in its compatibility with chondrocytes [16]. In an attempt to enhance its compatibility with osteoblasts, this study opted to modify PHBHHx biopolyester film through an aminolysis reaction using polyamidoamine dendrimer (PAMAM). PAMAM represents a novel class of multifunctional nano-polymer material [17]. It possesses favorable hydrodynamic properties, exhibits unique viscosity behavior, enables facile film formation, allows for easy modification, features nano-sized molecular solubility, and has a unique hollow structure. Owing to these remarkable attributes, it has emerged as an outstanding option for the development of novel drug-release systems [18]. As a nonsteroidal anti-inflammatory drug, ibuprofen has been widely applied for post-operative anti-inflammatory analgesia. Studies have shown that ibuprofen (IBU)-loaded scaffolds can enhance bone regeneration capacity [19]. In this study, a PHBHHx biopolyester film was aminolyzed using PAMAM. Subsequently, its properties were determined, encompassing surface characteristics, the quantity of cross-linked amino groups, hydrophilicity, ibuprofen release rates, and cytocompatibility. Meanwhile, osteoblasts were cultured on the biopolyester film to monitor their growth and investigate the compatibility between the biopolyester film and osteoblasts, thus devising a novel strategy for bone tissue engineering. This research is poised to offer crucial ideas and materials that will contribute to the application of biomaterials in bone tissue repair.

## 2. Results

### 2.1. Properties of PHBHHx Aminolyzed Biopolyester Films

**Figure 1:**
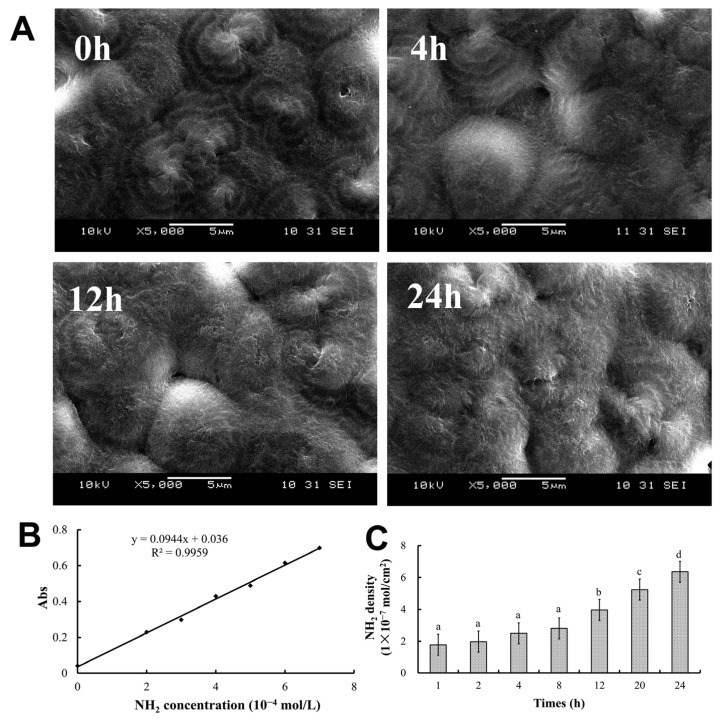
Properties of PHBHHx aminolyzed biopolyester films. (**A**) Surface morphology of PHBHHx biopolyester films with different ammonolysis times, observed using an SEM at 5000 times magnification. (**B**) Standard curve of the NH_2_ content using CEDA as a reference standard. (**C**) The density of NH_2_ in PHBHHx aminolyzed biopolyester films with different ammonolysis times. Different letters indicate significance by one-way ANOVA with Tukey’s post hoc test (*p* < 0.05).

Upon undergoing aminolysis by PAMAM, the surfaces of the PHBHHx films exhibited significant changes as the ammonolysis time lengthened (Figure 1A). Although spherical protrusions were also present on the surface of the non-aminolyzed PHBHHx film (at 0 h), they were far less conspicuous compared to those formed after ammonolysis. Meanwhile, as the ammonolysis time increased, the volume of the spherical protrusions on the film enlarged, multiple ‘hairs’ emerged, and the surface area of the film expanded.

In this study, PAMAM molecules were cross-linked to the PHBHHx surface through an aminolysis reaction. The degree of membrane aminolysis was analyzed using the ninhydrin reaction, in which the NH_2_ group reacts with indene and triketone to form a blue compound that can be measured using spectrophotometry at 540 nm.

The standard curve of NH_2_ content was plotted using CEDA as the standard (Figure 1B). A strong linear correlation can be observed between the absorbance value and the NH_2_ density shown, with the correlation coefficient exceeding 0.99. Based on this standard curve, the number of NH_2_ groups in the PHBHHx aminolyzed biopolyester film was determined. As depicted in Figure 1C, the number of NH₂ groups in the PHBHHx aminolyzed biopolyester film was dependent on the ammonolysis time. The NH_2_ density increased markedly until the ammonolysis time reached 12 h and continued to rise with the extension of the ammonolysis time, reaching its peak at 24 h. However, the results revealed that when the ammonolysis time exceeded 24 h, the membrane would become brittle and detach spontaneously. Therefore, in this study, the ammonolysis time of the membranes was confined within 24 h.

### 2.2. Wettability of PHBHHx Aminolyzed Biopolyester Films

The wettability of the PHBHHx aminolyzed biopolyester films was determined based on the water contact angle, which was tested using a contact angle meter. As shown in Table 1, the water contact angle of the PHBHHx membranes without PAMAM ammonolysis was 80.70 ± 2.06°. After PAMAM ammonolysis for 4 h, the water contact angle increased to 83.00 ± 1.56°. However, as the ammonolysis continued, the water contact angle gradually decreased. After 24 h of treatment, the water contact angle dropped to 68.60 ± 2.97°.

### 2.3. In Vitro Release of Ibuprofen on PHBHHx Aminolyzed Biopolyester Film

**Figure 2:**
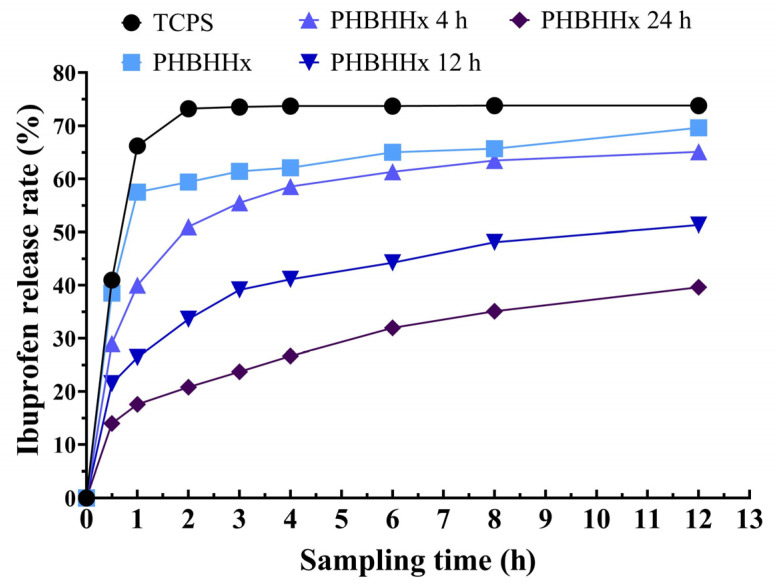
Ibuprofen release curve on PHBHHx aminolyzed biopolyester films.

Here, ibuprofen was used to investigate the drug-carrying capabilities of the PHBHHx aminolyzed biopolyester films. Ibuprofen was added dropwise and immobilized onto the PHBHHx aminolyzed biopolyester films in a dry state. After adding RPMI 1640 medium, ibuprofen was slowly released from the membrane into the medium. The in vitro release rate of ibuprofen was determined by measuring its content, thus enabling characterization of the drug-carrying properties of the PHBHHx aminolyzed biopolyester films. The results showed that the longer the membrane ammonolysis time was, the slower the release of ibuprofen was and the stronger the drug-carrying properties were (Figure 2).

### 2.4. Growth-Promoting Effect of PHBHHx Aminolyzed Biopolyester Films on Osteoblasts

**Figure 3:**
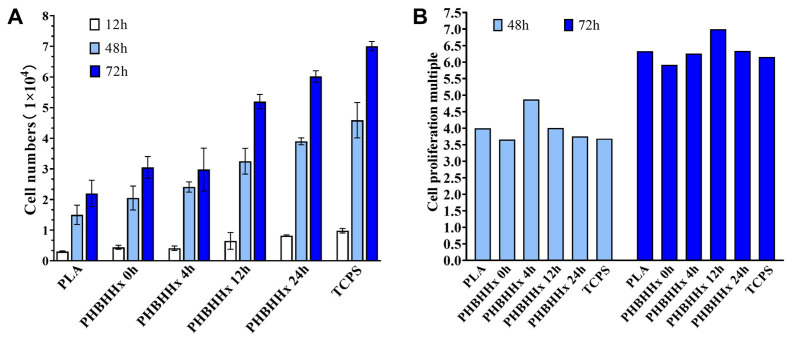
The cell numbers (**A**) and cell proliferation multiples (**B**) of osteoblasts on PHBHHx aminolyzed biopolyester films.

In this investigation, osteoblast cells were seeded on the PHBHHx aminolyzed biopolyester films and cultured for 12 h, 48 h, and 72 h. Subsequently, the number of cells and the proliferation rate were determined. The results shown in Figure 3 indicate that the osteoblasts grew well on the PHBHHx aminolyzed biopolyester films, with the cell number consistently increasing. Compared with the PLA membrane and the PHBHHx membrane without ammonolysis, the PHBHHx aminolyzed biopolyester film was more conducive to osteogenesis, as evidenced by the enhanced cell growth and number (Figure 3A). The proliferation multiples of osteoblasts cultured at 48 h and 72 h were determined (Figure 3B). When cultured for 48 h, the osteoblast proliferation rate on the PHBHHx biopolyester film with aminolysis for 4 h was the highest, but after 72 h of culture, the osteoblasts had the highest proliferation rate on the PHBHHx biopolyester film with aminolysis for 12 h. The results show that after aminolysis, the biopolyester films effectively promoted the growth of osteoblasts.

### 2.5. Compatibility Between PHBHHx Aminolyzed Biopolyester Film and Osteoblasts

**Figure 4:**
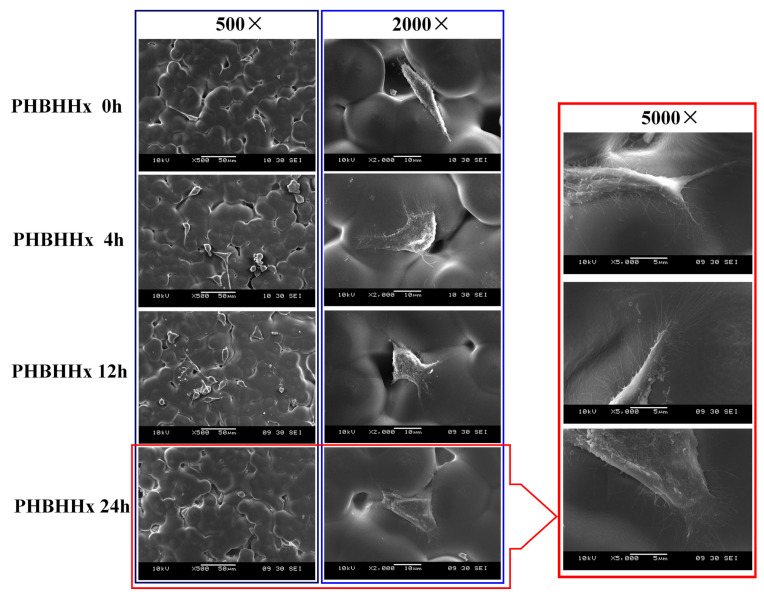
Cell morphology of osteoblasts on PHBHHx aminolyzed biopolyester film. The red border highlights the adhesion of osteoblasts on a PHBHHx biopolyester film that has been aminolyzed for 24 h, and these osteoblasts are magnified 5000 times.

To observe the adhesion and growth of osteoblasts on PHBHHx aminolyzed biopolyester film, osteoblasts that had been cultured on the PHBHHx aminolyzed biopolyester film for 36 h were imaged using SEM. The results are presented in Figure 4. It was found that the longer the ammonolysis time of the PHBHHx aminolyzed biopolyester film was, the better the adhesion and growth of osteoblasts were. Under 5000 times magnification, it was evident that the osteoblasts on the PHBHHx membrane after 24 h of ammonolysis were well spread, the connection between the cells and the biopolyester film was tight, and a large number of sturdy pseudopods were generated. This indicates that the PHBHHx membrane after 24 h of ammonolysis exhibited excellent compatibility with osteoblasts and could be utilized as a cell scaffold for culturing osteoblasts.

## 3. Discussion

The aminolysis process plays a crucial role in significantly modifying the surface morphology of PHBHHx biopolyester films by generating spherical protrusions and increasing the surface area, thereby altering the physical characteristics of the biopolyester films (Figure 1A). During this process, the primary amine groups on the dendrimer undergo substitution with other functional groups [17,18]. This substitution reaction is a key aspect of the overall transformation, and the aminolysis reaction effectively grafts NH_2_ groups onto the PHBHHx surface, which has been verified through spectrophotometric analysis. As the aminolysis time progressed, the density of NH_2_ groups increased in tandem, reaching its peak at exactly 24 h. This chemical modification enhances the biopolyester film’s functionality, strengthening its interaction with biomolecules and improving its compatibility with cells, allowing for more favorable interactions with the surrounding biological environment and potentially leading to better biological responses in various applications.

The water contact angle serves as a fundamental indicator of a biopolyester film’s hydrophilicity. Generally, a larger angle indicates a more hydrophobic biopolyester film, while a smaller angle indicates a more hydrophilic one. It has been demonstrated that the hydrophilicity of the PHBHHx membranes gradually increased after PAMAM ammonolysis for 12 h, and the wettability of the biopolyester film, as indicated by the water contact angle, exhibited a dynamic trend similar to that of PCL nanofibers undergoing direct aminolysis of PAMAM dendrimer [20]. The enhanced hydrophilicity after 12 h of aminolysis is of substantial importance, particularly for applications involving interaction with aqueous environments such as cell adhesion and drug delivery. A more hydrophilic surface facilitates better interaction with aqueous environments, enhancing cell adhesion by providing a favorable surface for cell attachment and improving drug delivery through better drug dispersion and solubility [21].

In the field of tissue engineering, nanomaterials have emerged as viable drug delivery vehicles [22]. In this study, PHBHHx biopolyester films conjugated with the nanomaterial PAMAM were shown to effectively carry and release ibuprofen. Interestingly, the release rate of ibuprofen was inversely proportional to the aminolysis time, suggesting that prolonged aminolysis improved the biopolyester film’s drug retention capability. This finding highlights the potential of PHBHHx biopolyester films as controlled drug-release systems, especially for sustained therapeutic applications. The underlying mechanism might be that extended aminolysis times lead to more complex surface modifications, providing favorable conditions for drug retention and enabling controlled drug release over an extended period, which is crucial for maintaining therapeutic efficacy.

In the specialized field of bone tissue engineering, the surface of bone implants must establish a close connection with living cells and possess osteogenic ability [23]. Aminolyzed PHBHHx biopolyester films have demonstrated a significant capacity to promote osteoblast adhesion and proliferation (Figure 3 and Figure 4). The biopolyester film with a 12 h aminolysis duration showed the highest proliferation rate at 72 h, indicating that this duration may provide an optimal environment for osteoblast growth at later stages. SEM observations revealed enhanced osteoblast adhesion and pseudopod formation on biopolyester films treated for 24 h, indicating excellent biocompatibility and potential as scaffolds for osteogenesis. These biopolyester films, through their modified properties, can create a conducive microenvironment for osteoblasts, similar to the natural extracellular matrix, facilitating bone tissue formation.

After modification, PAMAM molecules can bind more effectively, and their drug-loading capacity is enhanced [24]. In this study, the combination of PAMAM molecules with the PHBHHx membrane through ammonolysis is significant. This process alters the surface properties of the membrane, involving changes in surface chemistry, topography, and physical properties, and leads to the formation of a spatial structure by the PAMAM molecules that is more conducive to osteoblast attachment, thereby promoting their growth. The 24 h aminolysis biopolyester film exhibited optimal conditions for osteoblast growth, as evidenced by the tight cell–biopolyester film connections and the development of pseudopods. This indicates that such biopolyester films can serve as effective substrates for bone regeneration applications, with the 24 h time point seemingly representing an optimal balance between surface modification and biological compatibility, facilitating osteoblast growth and tissue regeneration.

The PHBHHx membrane offers unique advantages for bone tissue repair [25]. It can accurately provide spatial anchor points for the uniform growth of osteoblasts, serving as crucial foundations for their attachment and growth and thus significantly promoting osteoblast attachment and proliferation. It demonstrates excellent cytocompatibility, without inducing adverse reactions or harm to cells. When applied to the site of bone injury, it can efficiently exert its repair effectiveness by interacting positively with osteoblasts and supporting their growth and function. Notably, the PHBHHx aminolyzed biopolyester film is a biodegradable biomaterial. During patients’ bone injury repair, it degrades naturally, posing no harm to the human body. This natural degradation property avoids the need for a second-stage surgery to remove the membrane, significantly reducing patient pain [26]. However, it should be emphasized that the membrane was tested under in vitro conditions simulating the osteoblast growth environment, and although its good compatibility with osteoblasts has been verified, it still requires a series of rigorous clinical trials before being safely and effectively put into clinical use. Nevertheless, this membrane opens up a new strategic path for clinical bone tissue repair, representing an innovative approach with great potential for revolutionizing bone injury treatment and promoting bone tissue regeneration, although extensive clinical evaluation is necessary for full translation from laboratory to clinical practice.

## 4. Materials and Methods

### 4.1. Materials

Poly-3-hydroxybutyrate-co-3-hydroxyhexanoate (PHBHHx, Mw 400,000) consisting of 12% (mol) 3HHx was obtained from Lianyi Biotech Co., Ltd. (Guangzhou, China). PAMAM-G2-NH2 was purchased from Sigma-Aldrich (St. Louis, MO, USA). Dulbecco’s Modified Eagle Medium (DMEM) and RPMI 1640 culture media were purchased from Gibco-BRL (Gaithersburg, MD, USA), and fetal bovine serum (FBS) was purchased from Sijiqing Co., Ltd. (Hangzhou, China). Ibuprofen and ninhydrin were supplied by Juhua Pharmaceutical Factory (Hangzhou, China) and Sinopharm Chemical Reagent Co., Ltd. (Shanghai, China), respectively. All reagents were of analytical grade.

### 4.2. Preparation and Treatment of the Biopolyester Films

The films were prepared by dissolving 2.0 g of either PLA or PHBHHx in 100 mL of distilled chloroform; then, the mixtures were filtered through a 0.45 μm Millipore filter. A covered glass Petri dish (Φ30 mm) was used as the casting substrate. The mixtures were allowed to stand at room temperature (~20 °C) for three days, and then the films were dried in vacuum at 40 °C to remove trace solvent. The thickness of the resultant films was about 60 μm.

Next, the PHBHHx films were immersed in a methanol solution of PAMAM (G2.0) at a concentration of 10% (*w*/*v*) and allowed to react for the desired periods at 40 °C. Treated films were rinsed with 10^−4^ M hydrochloric acid for 1 h to remove unreacted PAMAM and then washed with cold distilled water for 24 h. The washed samples were dried under vacuum at 40 °C until a constant weight was achieved and stored in a desiccator before further use.

To observe the microstructures of the films, the films were placed in a sputtering device. They were coated with gold at a current of 15 mA for 1.5 min. Subsequently, the coated films were examined using a scanning electron microscope (JSM-6360LA, JEOL, Showima, Japan).

### 4.3. Ninhydrin Reaction

Ninhydrin reaction was employed to confirm the amination reaction and quantitatively ascertain the amounts of amino groups on the PHBHHx films. N-(β-aminoethyl)-γ-aminopropylmethyldimethoxyl silane (CEDA) purchased from Hangzhou Dadi Chemical Co., Ltd. (Hangzhou, China) served as the standard. Briefly, the aminolyzed film was immersed in 0.4 mL of a 1% (*w*/*v*) ninhydrin/methanol solution, followed by the addition of 1.6 mL of ethanol. The mixture was allowed to react at 80 °C for 15 min. Upon completion of the reaction, 6 mL chloroform was immediately added to dissolve the film. The absorbance at 450~700 nm of the resulting blue solution was recorded on a UV-Vis spectrophotometer (Beckman, Bria, CA, USA). A calibration curve was obtained to calculate the amino groups according to the absorbance at 550 nm.

### 4.4. Contact Angle Test

Wettability was examined by means of contact angle measurement. The contact angles were measured for the films on a contact angle meter (JY-82, Chengde Test-Machine Factory, Chengde, China). Redistilled water of approximately 10 μL was gently placed on the surface of the films. At least six readings on different parts of the films were averaged for data collection.

### 4.5. In Vitro Drug Release

In vitro release of ibuprofen from the PHBHHx films in RPMI 1640 medium was measured, with tissue culture polystyrene (TCPS) serving as the control. An ibuprofen–methanol solution (1 mL, 1 g/L) was added to the films or TCPS and then placed in the dark for 24 h. Afterwards, the films were dried in vacuum and RPMI 1640 culture medium was added. Subsequently, 0.5-milliliter samples were withdrawn at a designed interval, and fresh medium was replenished. The samples were dried and dissolved with methanol, and the concentration of ibuprofen was tested using high-performance liquid chromatography (HPLC) (Agilent 1100, Santa Clara, CA, USA) at 260 nm. The mobile phase was 35% acetonitrile in 0.1 M sodium acetate. The drug release rate was calculated as follows: divide the total amount of ibuprofen in the culture medium determined by HPLC by 1 mg, and then multiply the result by 100%.

### 4.6. Cell Attachment and Proliferation

The osteoblast cell line MC3T3 (Chinese Academy of Preventive Medical Sciences, Beijing, China) was cultured in DMEM supplemented with 10% FBS, 100 U/mL penicillin, and 100 mg/mL streptomycin. Cells were incubated at 37 °C in a 5% CO_2_ incubator, and the medium was refreshed every 2 days. When the cells reached confluence, they were harvested using 0.25% trypsin, followed by the addition of fresh culture medium to create a new single cell suspension for further inoculation.

The films were sterilized in 75% alcohol for 24 h and dried in a biological safety cabinet (Esco, Singapore) at room temperature. The osteoblast cells were seeded onto the films at a density of 1 × 10^4^/cm^2^ and then incubated at 37 °C in a 5% CO_2_ incubator. PLA films and tissue culture polystyrene (TCPS) (Costar, Cambridge, MA, USA) were used as controls. After incubation for 12 h, the films were transferred into new plates containing fresh medium, and the medium was refreshed every 2 days. Cells were harvested and enumerated at 12 h, 48 h, and 72 h post-seeding. MTT assay was performed to measure the activity of proliferation. Cells were treated with MTT at 48 h and 72 h and further incubated for 4 h. The culture medium was removed, and each well had 200 μL of acidic isopropanol to terminate the reaction. The absorbance was read using an ELISA plate reader (Multiskan FC, Shanghai, China) at 570 nm. The cell attachment and morphology were observed using a scanning electron microscope. Briefly, after the cells had been incubated for 36 h, the cell-seeded films were pretreated as follows: they were washed twice with cold phosphate-buffered saline (PBS) and immersed in PBS containing 2.5% glutaraldehyde (pH 7.4) at 4 °C for 12 h. They were then rinsed with PBS three times (15 min each) and dehydrated in increasing concentrations of ethanol (30%, 60%, 90%, 95%, 100%), followed by lyophilization. Finally, the dried samples were mounted on aluminum stumps, coated with gold in a sputtering device for 1.5 min at 15 mA, and examined under a scanning electron microscope (JSM-6360LA, JEOL, Showima, Japan).

### 4.7. Statistics

To determine whether the differences among multiple groups were significant (*p* < 0.05), a one-way ANOVA with Tukey’s post hoc test was utilized. Statistical figures were created using GraphPad Prism 9.0.

## 5. Conclusions

In this study, PHBHHx aminolyzed biopolyester films were fabricated and their properties as well as osteoblast compatibility were investigated. Through reaction with PAMAM, the membranes underwent significant surface alterations. Spherical protrusions emerged and grew in both number and size, accompanied by the formation of novel microstructures. The surface area expanded, and the density of amino groups increased, reaching a maximum at 24 h. However, when the ammonolysis time exceeded 24 h, the membrane became brittle. After 12 h of ammonolysis, the hydrophilicity of the membrane was enhanced. Prolonging the ammonolysis time led to a slower release rate of ibuprofen and improved drug-loading capacity. Osteoblasts exhibited favorable growth on the membrane; specifically, the membranes aminolyzed for 4 h and 12 h demonstrated the highest proliferation rates at 48 h and 72 h of culturing, respectively. The membrane with 24 h of ammonolysis displayed excellent biocompatibility. Overall, this membrane holds great promise for applications in tissue engineering. However, when the ammonolysis time exceeds 24 h, the membrane becomes brittle, which severely restricts its application. Therefore, it is essential to carry out combined treatment with compounds to enhance the membrane’s performance. In the future, research endeavors could be centered around further optimizing its properties and facilitating its translation into clinical practice.

## Figures and Tables

**Table 1:** Water contact angle of PHBHHx aminolyzed biopolyester films.

Item	Ammonolysis Time	Contact Angle (°)
PHBHHx0	0 h	80.70 ± 2.06 ^a^
PHBHHx4	4 h	83.00 ± 1.56 ^b^
PHBHHx12	12 h	78.67 ± 3.30 ^c^
PHBHHx24	24 h	68.60 ± 2.97 ^d^

Different letters indicate significance by one-way ANOVA with Tukey’s post hoc test (*p* < 0.05).

## Data Availability

The data supporting this article have been included as part of the main article and graphical abstract.

## Abbreviations

The following abbreviations are used in this manuscript:

CEDA	N-(β-aminoethyl)-γ-aminopropylmethyldimethoxyl silane
DMEM	Dulbecco’s Modified Eagle Medium
ECM	Extracellular matrix
FBS	Fetal bovine serum
HPLC	High-performance liquid chromatography
P3HB4HB	Poly-3-hydroxybutyrate-co-3-hydroxyvalerate
PAMAM	Polyamidoamine dendrimer
PHA	Polyhydroxyalkanoate
PHB	Poly-3-hydroxybutyrate
PHBHHx	Poly-3-hydroxybutyrate-co-3-hydroxyhexanoate
PHHx	Poly-3-hydroxyhexanoate
PHV	Poly-3-hydroxyvalerate
TCPS	Tissue culture polystyrene
